# Microbial signatures and host immune responses associated with the development of ventilator-associated pneumonia among patients with neurological injuries

**DOI:** 10.1128/spectrum.03193-25

**Published:** 2026-03-23

**Authors:** Leonardo Mancabelli, Emanuele Palomba, Federico Magni, Liliane Chatenoud, Ilaria Olgiati, Chiara Buracchi, Cristina Bugarin, Giuseppe Gaipa, Chiara Abbruzzese, Matteo Filippini, Andrea Forastieri, Roberto Fumagalli, Federico Geraldini, Edoardo Picetti, Leonardo Terranova, Rosanna Vaschetto, Tommaso Zoerle, Marco Ventura, Giuseppe Citerio, Andrea Gori, Alessandra Bandera, Laura Alagna, Francesca Turroni

**Affiliations:** 1 Department of Medicine and Surgery, University of Parma 9370, Parma, Italy; 2 Interdepartmental Research Centre "Microbiome Research Hub", University of Parma 9370, Parma, Italy; 3 Department of Infectious Diseases, Luigi Sacco Hospital 665657 , Milan, Italy; 4 Centre for Multidisciplinary Research in Health Science (MACH), University of Milan 9304, Milan, Italy; 5 NeuroIntensive Care Unit, Department Neuroscience, IRCCS Fondazione San Gerardo dei Tintori 9265 , Monza, Italy; 6 Istituto di Ricerche Farmacologiche Mario Negri IRCCS 574542 , Milan, Italy; 7 Tettamanti Center, Fondazione IRCCS San Gerardo dei Tintori 9265 , Monza, Italy; 8 Department of Anaesthesia, Critical Care and Emergency, Fondazione IRCCS Ca' Granda Ospedale Maggiore Policlinico 9339, Milan, Italy; 9 Department of Anesthesia, Intensive Care and Emergency, ASST Spedali Civili University Hospital 18515, Brescia, Italy; 10 Département Anesthésie Réanimation, Hospitalier Universitaire (CHU) Saint-Etienne 26926, Saint-Étienne, France; 11 Department of Medicine and Surgery, University of Milan-Bicocca 9305, Milan, Italy; 12 Department of Anesthesia and Intensive Care Medicine, Grande Ospedale Metropolitano Niguarda 9338, Milan, Italy; 13 Sant'Antonio Anesthesia and Intensive Care Unit, University-Hospital of Padua, Padua, Italy; 14 Department of Anaesthesia and Intensive Care, Parma University Hospital 18630 , Parma, Italy; 15 Internal Medicine Department, Respiratory Unit and Cystic Fibrosis Adult Center, Foundation IRCCS Cà Granda Ospedale Maggiore Policlinico, Milan, Italy; 16 Department of Anaesthesia and Intensive Care, Azienda Ospedaliero Universitaria Maggiore della Carità 9256, Novara, Italy; 17 Neuroscience Intensive Care Unit, Department of Anaesthesia and Critical Care, Fondazione IRCCS Ca' Granda Ospedale Maggiore Policlinico 9339, Milan, Italy; 18 Department of Pathophysiology and Transplantation, University of Milan 9304, Milan, Italy; 19 Laboratory of Probiogenomics, Department of Chemistry, Life Sciences and Environmental Sustainability, University of Parma 9370, Parma, Italy; 20 School of Medicine and Surgery, University of Milano-Bicocca 9305, Milan, Italy; 21 Department of Biomedical and Clinical Sciences "L. Sacco", University of Milan 9304, Milan, Italy; 22 Infectious Diseases Unit, Fondazione IRCCS Ca' Granda Ospedale Maggiore Policlinico 9339, Milan, Italy; Nova Southeastern University, Fort Lauderdale, Florida, USA

**Keywords:** human microbiota, ventilator-associated pneumonia, 16S rRNA, oropharyngeal swabs, endotracheal aspirate

## Abstract

Ventilator-associated pneumonia (VAP) remains a leading complication in mechanically ventilated patients, yet the contribution of the respiratory microbiota remains poorly understood. The PULMIVAP study is a multicenter, longitudinal cohort investigation of respiratory microbiota composition and host immune responses in critically ill adults intubated for non-pulmonary conditions. A total of 146 intubated adult patients were enrolled across eight Italian ICUs, forming matched groups of 73 with VAP and 73 without. Oropharyngeal swabs and endotracheal aspirates were collected at intubation and either at VAP diagnosis or at a matched point in controls for a total of 584 biological samples. Metataxonomic analyses revealed substantial temporal shifts in microbial communities across both upper and lower respiratory compartments, with a trend toward reduced microbial richness in patients who developed VAP. Several genera, such as *Corynebacterium,* were more abundant in no-VAP patients, whereas *Escherichia-Shigella* and *Peptoniphilus* were enriched in VAP samples. Cytokine-microbiota correlation analysis suggested a pro-inflammatory signature in VAP patients, with *Citrobacter* positively associated with IFN-γ and TNF-α, while several commensal genera were inversely correlated with inflammatory mediators. Additionally, taxa associated with VAP correlated with lower PaO_2_/FiO_2_ ratios, implicating them in disease severity. Consistently, several bacteria, such as *Corynebacterium*, appeared to be linked to better respiratory outcomes, suggesting protective or risk-associated microbial profiles. Overall, these findings highlight the complex interplay between microbial communities and mucosal immunity in the pathogenesis of VAP. The identification of condition-associated microbial and immunological signatures may inform future strategies for risk stratification and targeted prevention.

**IMPORTANCE:**

Ventilator-associated pneumonia (VAP) remains a major complication of mechanical ventilation, yet most microbiome studies have focused on late-stage infection or single airway compartments, limiting insight into early microbial dynamics associated with VAP risk. By longitudinally characterizing upper and lower airway microbiota before and during VAP development, this study provides new insights into microbial and immune patterns associated with susceptibility and disease severity in humans. These findings contribute to the current understanding of VAP pathogenesis by suggesting a role for early airway dysbiosis and local immune responses alongside clinical factors. Remarkably, the identification of taxa associated with risk or protection supports the potential for microbiota-informed monitoring and future risk stratification strategies during mechanical ventilation.

**CLINICAL TRIALS:**

This study is registered with ClinicalTrials.gov as NCT04849039.

## INTRODUCTION

Ventilator-associated pneumonia (VAP) is the most frequent nosocomial infection in intensive care units (ICUs) and remains a leading cause of morbidity and mortality among critically ill patients (1, 2). Despite the implementation of evidence-based prevention bundles (3), VAP continues to present major clinical challenges, largely due to difficulties in timely diagnosis and the complex, multifactorial nature of its pathogenesis. While factors such as microaspiration and colonization of the upper respiratory tract are known contributors (4), the biological processes that predispose patients to infection during mechanical ventilation remain incompletely understood.

In recent years, increasing attention has focused on the role of the respiratory microbiota in shaping host susceptibility to pulmonary infections (5). The upper airways host a diverse and dynamic microbial ecosystem that supports immune regulation and helps prevent overgrowth of pathogenic organisms (6, 7). This condition of microbial homeostasis, referred to as eubiosis, is maintained by a delicate balance that is particularly vulnerable in critical illness due to factors such as mechanical ventilation (MV), antibiotic exposure, systemic inflammation, and pulmonary complications (8–10). These perturbations may lead to dysbiosis, characterized by reduced microbial diversity and the expansion of opportunistic pathogens (11).

Emerging evidence suggests that such microbial alterations may increase the risk of VAP (12–14). However, most available studies have focused on lower airway samples collected after infection onset, limiting insight into early microbial shifts that may precede clinical manifestations (15, 16). As a result, it remains unclear whether microbiota alterations represent a consequence of infection or an antecedent state that contributes to susceptibility. This unresolved temporal relationship hampers the exploration of early predictive markers and preventive strategies during mechanical ventilation.

To address this knowledge gap, the PULMIVAP was designed to longitudinally characterize the respiratory microbiota in patients receiving MV for non-pulmonary indications. By integrating upper and lower airway sampling before and during VAP development, the study aims to identify early ecological patterns associated with susceptibility and disease severity and explore their potential relevance for respiratory dysfunction, as assessed by the PaO_2_/FiO_2_ ratio.

## MATERIALS AND METHODS

### Study design and setting

The PULMIVAP is an observational multicenter prospective cohort study designed with two main objectives: to characterize baseline features and longitudinal changes in the pulmonary microbiota that may influence the risk of VAP, and to identify clinical factors associated with the occurrence of a first VAP episode within 15 days of mechanical ventilation.

The study enrolled mechanically ventilated adult patients from eight ICUs in Northern Italy between September 2021 and April 2024 (Fig. S1a).

Inclusion criteria were intubation for non-pulmonary reasons, expected duration of mechanical ventilation >48 hours, absence of pneumonia at baseline, and limited prior antibiotic exposure (less than 72 hours before enrollment). We specifically restricted enrollment to patients intubated for non-pulmonary reasons in order to minimize potential confounding from pre-existing respiratory infections and to allow for a more accurate assessment of early microbiota changes associated with VAP development. Patients were followed prospectively for 15 days or until VAP diagnosis, ICU discharge, or death.

Clinical, microbiological, and therapeutic data were prospectively collected using a standardized Case Report Form via the REDCap platform. Follow-up continued until VAP diagnosis, ICU discharge, death, or up to 15 days of MV, whichever comes first. The VAP diagnosis was based on the attending physician’s clinical judgment, following the study consensus definition in accordance with international guidelines. Clinical parameters, including inflammatory progression, radiological findings, and pulmonary function deterioration, were evaluated daily (Fig. S1b). Oropharyngeal swab (OPS) and endotracheal aspirate (ETA) were longitudinally collected at baseline within the first 24 hours (*T*0) and at each follow-up day.

For the first aim, patients who developed ventilator-associated pneumonia within the first 15 days of intubation were matched 1:1 with patients who did not develop VAP during the same time window (Fig. S1c). Matching was performed within the center, based on reason for intubation (neurological vs non-neurological), sex, and age (±10 years). These variables were selected because they are potential factors associated with both VAP occurrence and microbiota composition (17–23). In total, 73 patients (among 148) with VAP were matched with 73 (among 258) patients without VAP (Fig. S1a and c). Oropharyngeal swab and endotracheal aspirate samples for microbiota analysis were those collected at baseline (*T*0), at the time of VAP (TVAP), diagnosis for VAP patients, and at the same MV day (i.e., TVAP) for matched non-VAP patients. In patients with VAP, microbiota was also analyzed on the MV day preceding VAP onset.

### DNA extraction

DNA was extracted from collected samples at Fondazione IRCCS Ospedale Maggiore Policlinico using ZymoBIOMICS DNA miniprep^©^. DNA quality and quantity were assessed by spectrophotometric measurements (260/280 and 260/230 ratios). Qualified DNA samples were sent to the Laboratory of Probiogenomics at the University of Parma for further molecular analysis.

### Microbiota identification by 16S rRNA gene amplification, sequencing, and data analysis

Partial 16S rRNA gene sequences were amplified from extracted DNA using primer pair Probio_Uni and Probio_Rev, targeting the V3 region of the 16S rRNA gene sequence (24). 16S rRNA gene amplification and amplicon checks were carried out as previously described (24). 16S rRNA gene sequencing was performed using a NextSeq (Illumina) according to the protocol previously reported (24).

Following sequencing, the .fastq files were processed using a custom script based on the QIIME software suite (25). Paired-end read pairs were assembled to reconstruct the complete Probio_Uni/Probio_Rev amplicons and filtered to retain sequences with a minimum length of 100 bp and an average quality score ≥ 25. Subsequently, potential human-derived sequences were removed by aligning the reads against a reference database of human genes using Bowtie2 (26, 27) and filtered out prior to downstream taxonomic analysis. Taxonomic classification was then performed using the BLAST-based classifier implemented in QIIME 2, aligned against the NCBI RefSeq 16S rRNA database (https://www.ncbi.nlm.nih.gov/refseq/targetedloci). Taxonomic profiles were generated by aggregating quality-filtered reads at the genus level for each sample, and all downstream analyses were performed on genus-level relative abundance data. Low-abundance taxa were filtered out using a relative abundance threshold of 0.01% and a minimum read count of 2 for taxonomic assignment.

### Mucosal immunity analysis

For cytokine quantification, endotracheal aspirates were thawed and centrifuged at high speed (15,000 rpm for 15 minutes, Centrifuge 5424 R, Eppendorf) to separate the supernatant from the cellular fraction. Following the manufacturer’s protocol, supernatants were analyzed using a Luminex MAGPIX multiplex platform. Briefly, magnetic beads pre-coated with specific capture antibodies targeting selected inflammatory mediators (TNF-α, IL-1α, IL-1β, IL-2, IL-4, IL-6, IL-8, IL-10, and IFN-γ) were incubated with the samples. Detection was achieved using biotinylated antibodies followed by streptavidin–phycoerythrin (PE) conjugate. Signal acquisition was performed on the MAGPIX system, identifying each analyte and quantifying PE intensity. Cytokine concentrations were calculated using the Quantist software (Bio-Techne).

### Statistical analysis

Diversity analyses were performed to characterize within-sample (alpha) and between-sample (beta) microbial variability. Biodiversity within a given sample (alpha diversity) was calculated using the species richness index, and group comparisons were assessed using Wilcoxon rank-sum tests with Benjamini-Hochberg correction for multiple testing. Between-sample diversity (beta-diversity) was assessed using Bray-Curtis dissimilarity and visualized through principal coordinates analysis (PCoA) with QIIME2 (25, 28). Differences in community composition were tested using permutational multivariate analysis of variance (PERMANOVA) (Adonis function, 999 permutations), with covariates including VAP status, sample type, hospital of origin, and prior antibiotic exposure (binary variable: yes/no).

Temporal intra-individual shifts were evaluated by calculating Bray-Curtis dissimilarity between *T*0 and TVAP samples within each subject. Moreover, Partitioning Around Medoids (PAM) clustering was applied to genus-level relative abundance profiles to identify community state types, with the optimal number of clusters determined via the elbow method.

Indicator species analysis was conducted using the generalized indicator value approach (IndVal.g) implemented in the indicspecies R package. Analyses were stratified by sample type and time point to account for compartment- and time-specific effects. Additional stratified analyses were performed to control for confounding by hospital of origin and prior antibiotic exposure. Only bacterial taxa with an average relative abundance ≥ 0.1% in at least one group were included. All *P*-values were adjusted for multiple testing using the Benjamini-Hochberg procedure.

Correlation analyses were performed using Spearman’s rank correlation. Correlations between cytokine concentrations and microbial genera were computed in ETA samples only, using taxa with relative abundance ≥ 0.1%. Correlations between VAP occurrence, PaO_2_/FiO_2_ ratio, and microbial genera were computed using taxa with prevalence > 20% and relative abundance ≥ 0.1%. All correlation *P*-values were adjusted for multiple comparisons using the Benjamini-Hochberg method.

Clinical and demographic variables were summarized according to their distribution: categorical variables were expressed as counts and percentages, while continuous variables were reported as means, medians, and interquartile ranges (*Q*1–*Q*3). Comparisons between VAP and no-VAP groups were performed using chi-square or Fisher’s exact tests for categorical variables and *t*-tests or Wilcoxon rank-sum tests for continuous variables.

Statistical analyses were conducted using R (version 4.5.1), SPSS (version 25), and SAS (version 9.4). All tests were two-tailed, and a *P*-value < 0.05 was considered statistically significant.

## RESULTS

### Study cohort and clinical features

Initially, 472 patients were enrolled. After excluding 51 patients with less than 3 days of follow-up, 11 patients with unreliable data on clinical outcomes, 3 patients with missing data on ICU vital status at discharge, and 1 patient who withdrew consent, a total of 406 patients were included in the final analysis (Fig. S1a). Among them, 148 (36.5%, 95% CI: 31.9–41.2) developed VAP within the first 15 days of MV (VAP patients), while 258 (63.5%) did not (no-VAP patients) (Fig. S1a and c).

The cumulative incidence of VAP within 15 days of intubation varied widely across centers, ranging from 8.3% (95% CI: 1.0–27.0) in Center G to 50.9% (95% CI: 38.1–63.6) in Center E (Table S1). This variation may partly reflect differences in patient management strategies between centers, rather than under-enrollment of no-VAP patients.

For microbiota analysis, a matched subset was selected from the overall cohort of VAP and no-VAP patients. Specifically, 73 patients were selected from the 148 patients who developed VAP and matched 1:1 with 73 patients selected from the 258 no-VAP patients within center, based on reason for intubation (neurological vs non-neurological), sex, and age (±10 years) (Fig. S1a and c). This matching strategy was implemented to ensure comparability between groups and enabled the comparison of OPS and ETA samples collected on the same MV days for VAP and no-VAP patients within each matched pair, namely at baseline (*T*0) and on the day of VAP diagnosis. Baseline (*T*0) sampling was performed as close as possible to the time of intubation and always within 24 hours of MV initiation.

Overall, 73 patients were included in each group, resulting in a total of 584 metataxonomic samples across all time points and both sample types (OPS and ETA) analyzed. After matching, no significant differences were observed between the 73 VAP and 73 no-VAP patients considered across most relevant clinical variables (Table S2). Similarly, there was no significant difference in survival rates between patients who developed VAP during the follow-up period and those who did not, but patients with VAP had a higher duration of MV (Table S3). More VAP recurrence was observed in the VAP group, although given the small numbers (4 vs 10), these results were not significant.

For comparison purposes, the setting and clinical characteristics of the entire cohort are reported in Table S4. Most patients were enrolled between 2021 and 2022 (270/406, 66.5%), with no difference between groups. MV was initiated in the hospital setting (emergency department or ICU) in approximately 72% of patients. Most patients (79%) were intubated for neurological reasons, with a significant difference between VAP and no-VAP patients (87.2% vs 74.5%, respectively; *P*-value = 0.002). VAP patients were more frequently male (102/148, 68.9%) compared to the no-VAP group (138/258, 53.5%; *P*-value = 0.002). The mean age of the overall cohort was 59.8 years; approximately 45% were within the normal BMI range, and 26% were smokers, with no marked differences between groups.

Regarding patient comorbidities, more than half (56%) had at least one of the conditions studied (diabetes mellitus, liver failure, chronic inflammatory/autoimmune disease, respiratory diseases, cardiovascular disease, chronic renal failure, including dialysis, and immunocompromised status), with a comparable distribution between groups. VAP patients had lower Glasgow Coma Scale (GCS) scores at intubation: 62.8% (93/148) had a score between 3 and 8, compared to 53.1% (137/258) in the no-VAP group (*P*-value = 0.010), suggesting a higher risk of poor outcomes. However, APACHE II scores were similar between the groups. Respiratory parameters at intubation (PEEP, FiO_2_, and PaO_2_/FiO_2_) were similarly distributed between groups.

### Microbiota profiling and bacterial richness

A total of 584 biological samples were included in the metataxonomic analyses, obtained from 73 no-VAP patients at time points *T*0 and TVAP, and 73 VAP patients collected at the same time point. Samples were obtained from ETA and OPS for each time point.

Microbial composition was assessed through 16S rRNA gene sequencing, yielding a total of 25,542,603 raw reads. After quality filtering, chimera removal, and read denoising, 18,605,901 high-quality reads were retained, with a mean read depth of 25,488 ± 12,078 per sample (Table S5). The taxonomic assignment allowed reliable classification at the genus level (Table S6) and subsequent diversity analyses.

**Fig 1:**
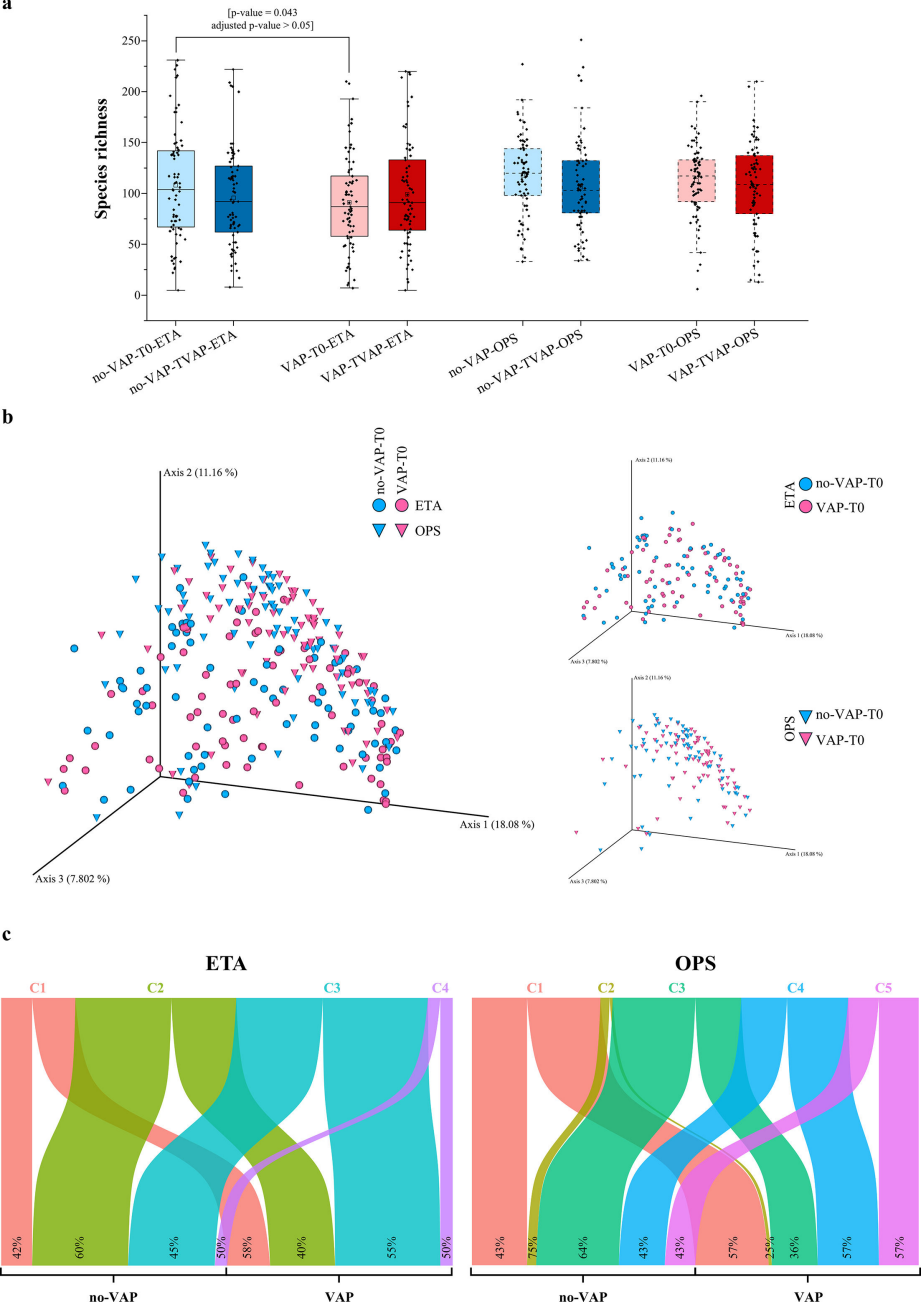
Temporal dynamics and structural diversity of the respiratory microbiota in mechanically ventilated patients. (**a**) Box-and-whisker plots showing microbial species richness across sample types (ETA and OPS), time points (baseline [*T*0] and follow-up [TVAP]), and VAP status (no-VAP vs VAP). Boxes represent the interquartile range (IQR), whiskers indicate 1.5× IQR, and horizontal lines denote the median values. (**b**) Principal coordinates analysis of baseline (*T*0) genus-level microbial profiles based on Bray-Curtis dissimilarity. Samples are color-coded by VAP status (blue, no-VAP; violet, VAP) and shaped by sample type (circles, ETA; triangles, OPS). Side panels display stratified views for each sample type. PCoA ordination is shown for descriptive purposes; statistical significance of group differences was assessed using PERMANOVA and is reported in the Results section. (**c**) Sankey diagrams illustrating intra-patient transitions of microbial community clusters from *T*0 to TVAP in the VAP and no-VAP groups. Colors represent cluster assignments derived from PAM analysis. Flow widths and percentage labels indicate the proportion of samples maintaining or shifting cluster membership over time.

The alpha diversity analysis, measured as species richness, revealed early differences in microbial complexity between VAP and no-VAP groups (Fig. 1a). At *T*0, ETA samples from no-VAP patients exhibited a higher average richness of 108 ± 55 compared to 96 ± 48 from VAP patients (*P*-value = 0.043, adjusted *P*-value > 0.05), suggesting a trend toward reduced microbial complexity in patients who later developed VAP (Fig. 1a). Over time, this difference appeared to diminish, with a general decrease in richness observed across both groups, albeit not reaching statistical significance (Fig. 1a). These findings may reflect the impact of intubation and mechanical ventilation on the upper and lower respiratory microbiota.

Additionally, to explore the relationship between oral and pulmonary microbial communities, we quantified the proportion of bacterial genera shared between ETA and OPS samples at each time point as a measure of compartmental overlap (Fig. S2a). On average, 46% ± 16% and 50% ± 20% of genera detected in ETA samples were also present in the corresponding OPS samples in no-VAP patients at *T*0 and TVAP, respectively. Similarly, in VAP patients, 45% ± 18% and 49% ± 19% of genera were shared at *T*0 and TVAP (Fig. S2a). These results suggest a consistent, partial overlap between the two compartments across groups and time points. However, no significant differences were observed in the proportion of shared taxa when stratified by VAP status or sampling time.

### Intra-individual analyses and possible microbial shifts

To investigate whether microbial composition at the time of intubation could be indicative of future VAP development, beta diversity analyses were performed at baseline, focusing exclusively on ETA and OPS samples collected at time point *T*0. Community dissimilarities were calculated using Bray-Curtis distances and visualized through PCoA (Fig. 1b). Permutational multivariate analysis of variance (using the Adonis function) was employed to test for statistically significant differences in microbial community structure. In addition to VAP status, we included other clinically relevant covariates such as sample type, i.e., ETA and OPS, hospital of origin, and antibiotic exposure at intubation. The analyses revealed that VAP status (VAP vs no-VAP) was significantly associated with microbial composition (*P*-value = 0.027), although it explained a very limited portion of the overall variance (*R*^2^ = 0.0066). Antibiotic exposure at intubation was also associated with modest differences (*P*-value = 0.008, *R*^2^ = 0.0127). Notably, sampling type and hospital of origin showed stronger associations (*R*^2^ = 0.0471 and 0.0389, respectively, both *P*-value < 0.001), suggesting that anatomical site and the hospital may substantially influence community structure more than disease progression alone. Altogether, these findings indicate that the early microbial landscape is shaped by multifactorial influences, with only subtle stratification linked to future VAP onset.

To evaluate intra-patient temporal variation in ETA and OPS samples, Bray-Curtis dissimilarity was computed between *T*0 and TVAP for each individual. This approach enabled the assessment of whether the onset of VAP was associated with more pronounced shifts in microbial community structure over time (Fig. S2b). The analysis revealed substantial temporal variability between baseline and follow-up samples across all conditions, including both VAP and no-VAP groups, and in both ETA and OPS samples (Fig. S2b). However, no consistent or statistically significant differences in dissimilarity were observed between patients with and without VAP (Fig. S2b). These findings likely reflect the combined effects of mechanical ventilation, antibiotic administration, and intrinsic sampling variability, rather than being directly attributable to disease progression. Temporal shifts in the microbial composition were previously observed in both lower and upper respiratory tracts among mechanically ventilated patients (14, 29), confirming the role of intubation in these dynamic changes. However, the role of intubation in the occurrence of VAP needs to be clarified.

### Baseline microbial community patterns and potential associations with VAP susceptibility

To further assess whether microbial community profiles exhibited latent patterns related to VAP development at baseline, we performed unsupervised clustering using Partitioning Around Medoids on genus-level relative abundance profiles of ETA and OPS samples collected at *T*0. The optimal number of clusters was determined using the elbow method, identifying four distinct microbial clusters for ETA samples and five for OPS (Fig. 1c; Table S7). Although chi-squared tests did not reveal statistically significant associations between cluster membership and VAP status (ETA *P*-value = 0.366 and OPS *P*-value = 0.172), some compositional trends were observed (Fig. 1c). For ETA samples, cluster 2, which is characterized by a diverse composition, including *Staphylococcus* and *Escherichia-Shigella*, was more frequently associated with no-VAP patients (prevalence of 60%), with no taxa exceeding 5% relative abundance. On the other hand, cluster 3, which is characterized by a higher prevalence of *Streptococcus*, *Haemophilus*, *Prevotella*, *Neisseria*, and *Gemella*, was more commonly found in the VAP group (prevalence of 55%), potentially reflecting a higher presence of these bacteria potentially involved in VAP occurrence (12, 30, 31). These microorganisms are frequently implicated in the pathogenesis of ventilator-associated pneumonia in patients who have been intubated for neurological reasons (32). Changes in commensal composition with predominance of potentially pathogenic agents could influence VAP occurrence, as previously highlighted (33).

Similarly, in OPS samples, cluster 3, which is enriched with *Prevotella*, *Streptococcus*, *Veillonella*, and *Segatella*, was more common in no-VAP patients (prevalence of 64%). Clusters 4 and 5, which showed higher proportions in the VAP group (prevalence of 57% in both), included a predominance of *Streptococcus* in cluster 4 and a mix of *Neisseria*, *Streptococcus*, *Prevotella*, *Haemophilus*, and *Porphyromonas* in cluster 5, again raising the possibility of the involvement of bacteria potentially involved in VAP development (12, 30, 32). Despite the lack of statistical significance, these compositional trends may capture baseline heterogeneity in microbial community structure, which could plausibly modulate susceptibility to subsequent perturbations such as VAP. At the same time, the absence of a clear association between PAM clusters and VAP status indicates that these results should be interpreted as exploratory and potentially influenced by methodological factors rather than reflecting stable disease-associated microbial states.

### Identification of bacterial VAP biomarkers

We performed an analysis of indicator bacterial species based on permutation testing to investigate taxonomic signatures associated with the onset of VAP. This method identifies frequent and abundant microbial taxa within a specific condition (VAP or no-VAP), capturing ecologically meaningful associations. The analysis was restricted to microbial genera with a relative abundance greater than 0.1% in at least one group to reduce noise from rare taxa.

Given the influence of sample type, hospital of origin, and prior antibiotic exposure observed in beta-diversity analyses, we stratified the comparisons accordingly. Indicator microbial species analyses were conducted independently for each sample type and at each time point, allowing for the detection of compartment- and time-specific patterns. This strategy also reflects the considerable temporal variability in respiratory microbiota, which could complicate model interpretability and reduce statistical robustness in the context of limited sampling time points and cohort size.

In addition, parallel indicator species analyses were performed separately for the hospital of origin and prior antibiotic exposure to minimize potential confounding effects. Bacterial taxa significantly associated with these factors were excluded from the primary comparison between no-VAP and VAP samples to ensure specificity.

**Fig 2:**
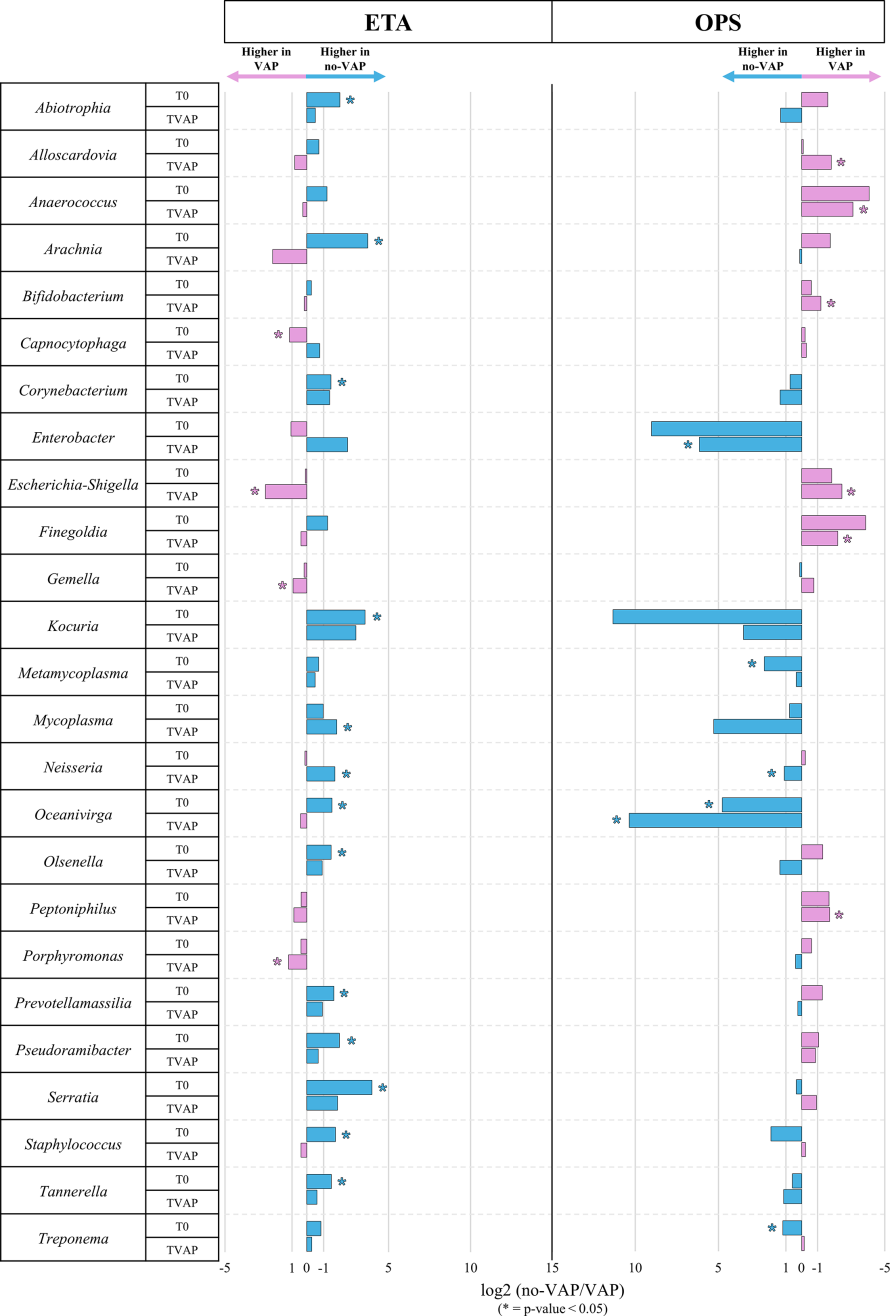
Differentially abundant bacterial genera associated with VAP and no-VAP status across sample types and time points. Bar plots showing genera with significant differential abundance between no-VAP (blue) and VAP (violet) groups in ETA (left) and OPS (right) samples at baseline (*T*0) and follow-up (TVAP). The horizontal axis represents the log_2_ ratio of relative abundances (log₂[no-VAP/VAP]), indicating the direction and magnitude of difference. Asterisks denote genera identified as significant (*P*-value < 0.05, Benjamini-Hochberg adjusted) using the IndVal.g permutation-based indicator species analysis.

After filtering, the statistical analyses revealed that 25 microbial genera were significantly associated (*P*-value < 0.05) with at least one group in a specific sample type and time point (Fig. 2). Notably, *Corynebacterium*, *Kocuria*, *Metamycoplasma*, *Mycoplasma*, and *Tannerella* were consistently more abundant in no-VAP patients, both at baseline and follow-up, across ETA and OPS samples. In contrast, *Escherichia-Shigella* and *Peptoniphilus* are enriched in VAP patients under the same conditions. The persistent presence of commensal genera such as *Corynebacterium* in non-VAP patients may reflect a protective ecological role. Previous studies suggest that *Corynebacterium* spp. can inhibit the growth of opportunistic pathogens, such as *Streptococcus pneumoniae* and *Staphylococcus aureus*, thereby contributing to mucosal homeostasis and protection against infection (34, 35). Conversely, the increased abundance of *Escherichia-Shigella* and *Peptoniphilus* in VAP patients could indicate a state of early dysbiosis associated with inflammation and greater susceptibility to pneumonia (12, 36, 37).

*Gemella* and *Porphyromonas* emerged as genera of particular interest in ETA samples from VAP patients. Both are primarily associated with the oral microbiota (38, 39) and often occur in polymicrobial communities in the upper airways (12, 40, 41). Their selective enrichment in lower airways, without corresponding increases in OPS samples (Fig. 2), suggests possible translocation or local expansion following intubation. Indeed, endotracheal intubation can compromise anatomical barriers, facilitating the migration of oral taxa into the lungs (11, 42). Notably, *Gemella* and *Porphyromonas* have been implicated in aspiration pneumonia and polymicrobial lung infections, particularly in immunocompromised or critically ill patients (43–45).

Altogether, these findings support the existence of early and potentially persistent microbial signatures associated with VAP susceptibility, highlighting the importance of controlling for confounders in microbiome studies involving heterogeneous ICU populations.

### Correlation between ETA microbiota and mucosal immunity during mechanical ventilation

To investigate potential interactions between the airway microbiota and local immune responses, Spearman correlation analysis was performed between cytokine levels (Table S5 ) and microbial relative abundances in ETA samples.

Due to the limited availability of biological material, cytokine measurements were only available for a subset of patients (*n* = 43 and 53 for VAP at *T*0 and TVAP, respectively; *n* = 46 and 45 for no-VAP). Cytokine and microbiota analyses were performed on ETA samples collected at the same clinical time point, and only paired samples were included in correlation analyses. Despite this limitation, which prevents the results from being generalized, the analysis provided preliminary insights into the complex interplay between the lower respiratory tract microbiota and the mucosal immune environment during mechanical ventilation. Overall, analysis of the cytokine profile during MV revealed that VAP expresses more pro-inflammatory cytokines (e.g., IL-1α) at intubation, while all patients show an increase in IL-1β production during mechanical ventilation compared to intubation (Fig. S3). Moreover, only microbial taxa with a relative abundance greater than 0.1% in at least one sample were considered.

The statistical analysis identified a total of 73 significant correlations (false discovery rate-adjusted *P*-value < 0.05), of which 12 were positive, and 61 were negative (Table 1). Notably, the *Citrobacter* genus was positively associated with several pro-inflammatory cytokines, including IFN-γ, IL-2, IL-4, and TNF-α, potentially reflecting a role in immune activation (46). In contrast, most correlations were negative, particularly involving bacterial taxa such as *Rothia*, *Veillonella*, *Sutterella*, *Lacticaseibacillus*, and *Actinomyces*, which were inversely associated with inflammatory mediators like IL-1β, TNF-α, and IL-10. These associations may suggest a more regulated or suppressed immune state or reflect a reduced inflammatory response in the presence of these bacteria (47).

**TABLE 1:** Significant correlations between bacterial genera and cytokine levels in ETA samples

Var1	Taxa	Pearson correlation	*P*-value (adjusted BH)
IFN-γ	*Citrobacter*	0.321	0.000
IFN-γ	*Bacillus*	0.219	0.032
IL-10	*Rothia*	−0.234	0.018
IL-10	*Veillonella*	−0.219	0.032
IL-10	*Sutterella*	−0.206	0.048
IL-10	*Limosilactobacillus*	−0.205	0.050
IL-1α	*Moraxella*	−0.255	0.008
IL-1α	*Hallella*	−0.247	0.011
IL-1α	*Mitsuokella*	−0.240	0.015
IL-1α	*Rothia*	−0.233	0.019
IL-1α	*Actinomyces*	−0.230	0.022
IL-1α	*Wolinella*	−0.224	0.026
IL-1α	*Abiotrophia*	−0.223	0.027
IL-1α	*Scardovia*	−0.221	0.030
IL-1α	*Leptotrichia*	−0.217	0.033
IL-1α	*Veillonella*	−0.216	0.036
IL-1β	*Limosilactobacillus*	−0.315	0.001
IL-1β	*Lacticaseibacillus*	−0.308	0.001
IL-1β	*Rothia*	−0.273	0.004
IL-1β	*Latilactobacillus*	−0.269	0.005
IL-1β	*Ruminococcus*	−0.268	0.005
IL-1β	*Anaerococcus*	−0.257	0.008
IL-1β	*Scardovia*	−0.255	0.008
IL-1β	*Eubacterium*	−0.245	0.012
IL-1β	*Veillonella*	−0.243	0.013
IL-1β	*Sneathia*	−0.235	0.018
IL-1β	*Lachnoanaerobaculum*	−0.226	0.025
IL-1β	*Gardnerella*	−0.224	0.026
IL-1β	*Raoultella*	−0.220	0.030
IL-1β	*Finegoldia*	−0.219	0.032
IL-1β	*Sutterella*	−0.214	0.037
IL-1β	*Actinomyces*	−0.213	0.039
IL-1β	*Schaalia*	−0.206	0.049
IL-1β	*Alloprevotella*	0.263	0.006
IL-2	*Rothia*	−0.261	0.006
IL-2	*Actinomyces*	−0.238	0.016
IL-2	*Abiotrophia*	−0.231	0.021
IL-2	*Anaeroglobus*	−0.228	0.023
IL-2	*Filifactor*	−0.214	0.038
IL-2	*Citrobacter*	0.320	0.000
IL-4	*Peptostreptococcus*	−0.212	0.040
IL-4	*Alloprevotella*	−0.208	0.046
IL-4	*Lacticaseibacillus*	0.276	0.003
IL-4	*Citrobacter*	0.347	0.000
IL-6	*Alloprevotella*	−0.219	0.032
IL-6	*Arachnia*	0.205	0.050
IL-6	Lachnospiraceae family	0.210	0.043
IL-6	*Lacticaseibacillus*	0.215	0.036
IL-6	*Lachnoanaerobaculum*	0.218	0.032
IL-8	*Gemella*	0.210	0.043
TNF-α	*Prevotella*	−0.297	0.001
TNF-α	*Veillonella*	−0.287	0.002
TNF-α	*Selenomonas*	−0.284	0.002
TNF-α	*Moraxella*	−0.283	0.003
TNF-α	*Peptococcus*	−0.281	0.003
TNF-α	*Eubacterium*	−0.274	0.004
TNF-α	*Kocuria*	−0.269	0.005
TNF-α	*Schaalia*	−0.264	0.006
TNF-α	*Scardovia*	−0.246	0.012
TNF-α	*Actinomyces*	−0.244	0.013
TNF-α	*Olsenella*	−0.241	0.014
TNF-α	*Mitsuokella*	−0.232	0.020
TNF-α	*Dialister*	−0.224	0.026
TNF-α	*Acinetobacter*	−0.221	0.029
TNF-α	*Treponema*	−0.214	0.037
TNF-α	*Corynebacterium*	−0.212	0.040
TNF-α	*Limosilactobacillus*	−0.211	0.042
TNF-α	*Lachnoanaerobaculum*	−0.211	0.042
TNF-α	*Rothia*	−0.209	0.044
TNF-α	*Abiotrophia*	−0.207	0.048
TNF-α	*Anaeroglobus*	−0.206	0.048
TNF-α	*Porphyromonas*	−0.206	0.049
TNF-α	*Citrobacter*	0.321	0.000

Interestingly, many of the negatively correlated taxa are common members of the oral microbiota, raising the notion that translocated oropharyngeal bacteria might colonize the lower airways without necessarily eliciting a strong pro-inflammatory response, especially in a population of critically ill but non-pneumonic patients, who were intubated for non-respiratory conditions (43–45).

These findings support the assumption that specific members of the airway microbiota may be closely linked to the local immune landscape in intubated patients. While it remains unclear whether these microbial communities actively modulate the mucosal immune response or are shaped by the host’s immunological status, the observed correlations highlight a potentially dynamic, bidirectional relationship. This interplay may have important implications for susceptibility to respiratory complications, including the development of ventilator-associated pneumonia, and warrants further investigation in larger, longitudinally sampled cohorts.

### Protective and risk-associated microbial signatures in VAP and respiratory function

To further explore the relationship between the respiratory microbiota and clinical outcomes, we focused on assessing potential associations between microbial communities, the development of VAP, and disease severity, as measured by the PaO_2_/FiO_2_ ratio (Table S5), which is a marker of respiratory function impairment.

This analysis was performed considering all available samples (ETA and OPS) jointly, including an additional time point collected 48 hours before VAP onset in affected patients. This approach aimed to enhance statistical power and provide a more comprehensive understanding of the microbial taxa potentially involved in the development or severity of VAP. Importantly, the correlation analysis was restricted to taxa with a prevalence greater than 20% and an average relative abundance above 0.1%, thereby reducing noise from low-abundance genera and focusing on ecologically relevant taxa.

**Fig 3:**
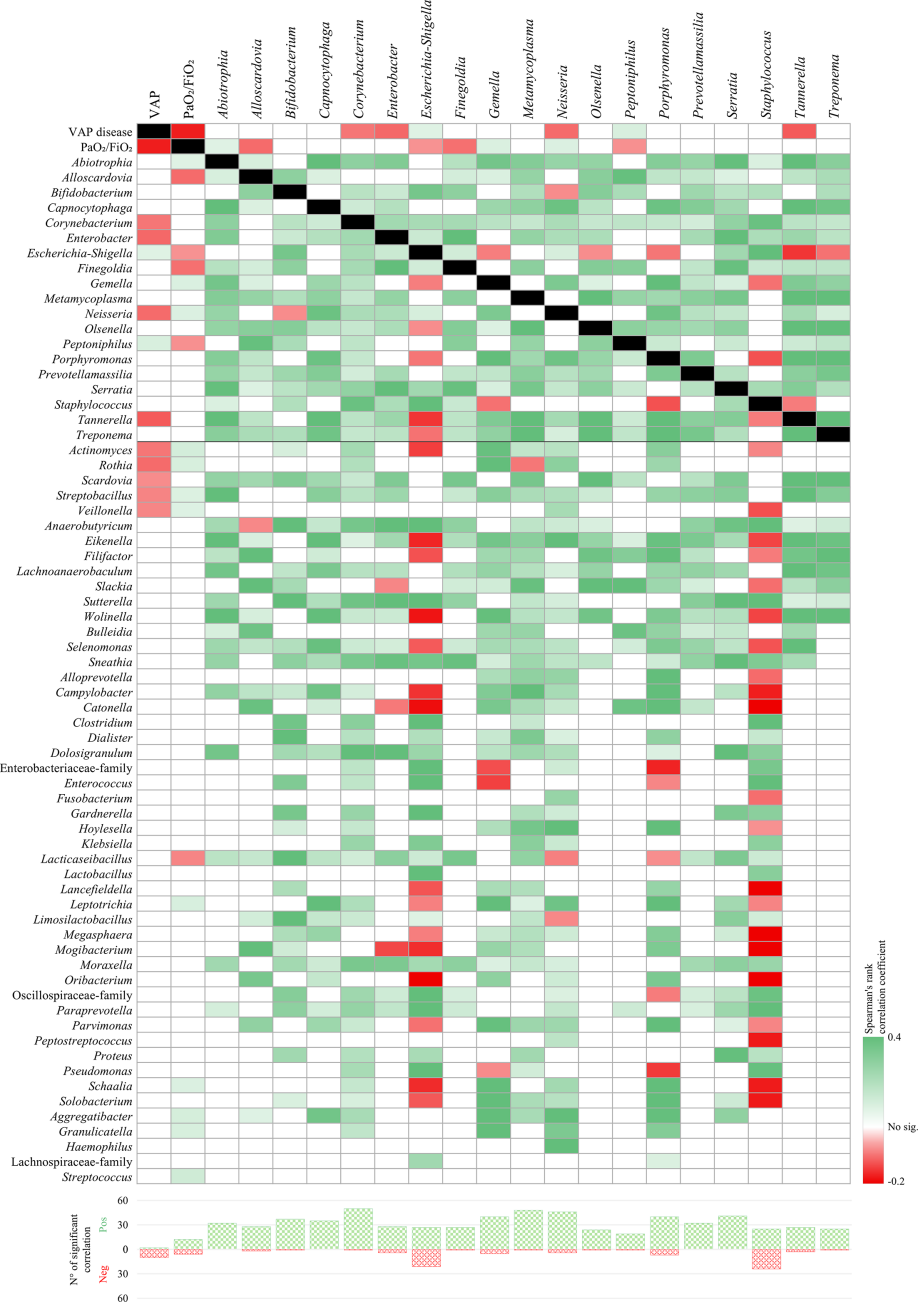
Correlations between clinical parameters (VAP occurrence and PaO_2_/FiO_2_ ratio) and microbial taxa. The heatmap displays Spearman correlation coefficients calculated among clinical variables (VAP occurrence and PaO_2_/FiO_2_ ratio) and microbial taxa. Rows include, in order: the 2 clinical variables (VAP occurrence and PaO_2_/FiO_2_ ratio), the 19 bacterial taxa previously identified as significant in the taxonomic analysis (filtered for prevalence > 20% and relative abundance average > 0.1%), and additional taxa that were significantly correlated with either VAP or PaO_2_/FiO_2_ in this analysis, also filtered for prevalence and abundance as above. Columns display only the 21 primary variables of interest: the 2 clinical variables and the 19 significant taxa from the previous taxonomic analysis. Cells are color-coded to indicate the direction and significance of the correlations: red indicates significant negative correlations, green indicates significant positive correlations, white denotes non-significant correlations, and black marks self-comparisons. The bar plot at the bottom represents the total number of significant correlations identified for each column.

In relation to VAP occurrence, the analysis confirmed the findings obtained in the previous taxonomic comparisons, revealing several bacterial taxa that may exert a protective effect against VAP development (Fig. 3). Genera such as *Corynebacterium* and *Tannerella*, alongside other taxa, including *Enterobacter*, *Actinomyces*, *Rothia*, *Scardovia*, *Streptobacillus*, and *Veillonella*, were found to be negatively associated with VAP. Conversely, *Escherichia-Shigella* and *Peptoniphilus* showed a positive association with VAP occurrence, indicating a link between their increased abundance and susceptibility to infection (48, 49).

Assessment of respiratory function using the PaO_₂_/FiO_₂_ ratio revealed that several of the same taxa also showed significant correlations with this parameter. *Actinomyces*, *Rothia*, *Streptobacillus*, and *Veillonella* were positively correlated with higher PaO_2_/FiO_2_ values, suggesting a potential role in preserving respiratory function and mitigating disease severity (42, 47, 48). In contrast, *Escherichia-Shigella* and *Peptoniphilus* were correlated with lower PaO_₂_/FiO_₂_ values, consistent with a potential contribution to respiratory dysfunction (48, 49).

Notably, other genera, such as *Alloscardovia*, *Finegoldia*, and *Lacticaseibacillus*, also showed a correlation with lower PaO_2_/FiO_2_ values but not with VAP occurrence, suggesting that specific taxa may be explicitly linked to impaired respiratory function rather than directly to VAP development. These findings support the hypothesis that specific microbial signatures may contribute to both susceptibility to VAP and the severity of respiratory compromise in mechanically ventilated patients, in line with previous reports suggesting protective roles for genera such as *Corynebacterium* and *Veillonella* in maintaining respiratory microbial balance and mucosal immunity (50, 51). Conversely, *Escherichia-Shigella* has been repeatedly associated with respiratory dysbiosis and worse clinical outcomes in critically ill patients (49, 52).

Furthermore, to explore microbial interaction patterns, correlations among taxa identified as significant in the preceding analyses (Fig. 2) were investigated. Significant positive correlations were found between specific taxa (Fig. 3), possibly reflecting cooperative interactions within the respiratory microbiota ecosystem (53). In contrast, *Escherichia-Shigella* and *Staphylococcus* exhibited numerous negative correlations, suggesting their potential role in altering microbial networks and promoting microbial dysbiosis. Notably, several of these negative correlations involved taxa previously proposed as beneficial, such as *Tannerella*, *Actinomyces*, and *Veillonella*, highlighting the complex and context-dependent nature of microbial interactions in the respiratory tract (53).

## DISCUSSION

Using a standardized sampling protocol and a matched-cohort design, this study represents one of the most extensive multicenter efforts to longitudinally characterize the upper and lower airway microbiota in mechanically ventilated patients intubated for non-pulmonary reasons at risk of VAP.

Metataxonomic analyses revealed substantial inter- and intra-patient variability in respiratory microbiota composition over time and across sampling compartments, highlighting the complexity of microbial dynamics during mechanical ventilation and the challenges of identifying uniform microbial signatures across heterogeneous ICU populations.

Most previous investigations have focused on lower airway samples collected after infection onset, thus limiting insight into early microbial shifts that may precede clinical manifestations. By contrast, the present longitudinal design enabled the capture of pre-infection dynamics and their association with host respiratory function.

At baseline, a modest reduction in microbial richness was observed in VAP patients compared to matched no-VAP controls. Interestingly, partial stabilization of microbial profiles was observed at later time points, suggesting a potential convergence toward a ventilator-associated ecological state. Moreover, Bray-Curtis dissimilarity analyses revealed temporal shifts within individuals, independent of VAP status, highlighting the high variability of respiratory microbiota communities, which may also be influenced by mechanical ventilation, antibiotic exposure, and ICU-related factors.

The consistent overlap of bacterial genera between oropharyngeal and bronchial samples supports the existence of an oral-lung axis, with intubation potentially facilitating microbial migration. However, PERMANOVA analyses indicated that OPS and ETA retained distinct compositional and ecological features, indicating that upper and lower airway microbiota represent partially overlapping but distinct ecological compartments. The proportion of shared bacterial taxa remained stable across time points and conditions, suggesting that translocation alone is unlikely to drive disease progression. Instead, the expansion or persistence of specific oral-origin taxa in the lower airways may depend on host factors and local ecological dynamics, with reduced ability to maintain equilibrium within commensal microorganisms.

Furthermore, several genera showed condition-associated trends. *Corynebacterium*, *Kocuria*, *Metamycoplasma*, and *Mycoplasma* were more prevalent in no-VAP patients, possibly reflecting protective roles via colonization resistance or immune modulation. Conversely, *Escherichia-Shigella*, *Peptoniphilus*, *Gemella*, and *Porphyromonas* were enriched in VAP samples, consistent with a potential role in early dysbiosis or opportunistic pathogenicity. Nevertheless, these associations were inconsistent across individuals and time points, reflecting the multifactorial nature of microbial interactions in critical illness.

Exploratory cytokine–microbiota analyses suggested potential associations between microbial taxa and local immune responses during mechanical ventilation. Positive correlations between *Citrobacter* and pro-inflammatory mediators were observed, whereas several oral-associated genera, including *Veillonella*, *Rothia*, and *Lacticaseibacillus*, showed inverse correlations with inflammatory markers. These findings should be interpreted cautiously, as cytokine data were available only for a subset of patients and the analyses were exploratory in nature.

In addition, correlation analyses incorporating the PaO_₂_/FiO_₂_ ratio further suggested that certain taxa may be associated not only with VAP occurrence but also with the severity of respiratory dysfunction. Genera such as *Corynebacterium*, *Tannerella*, and *Veillonella* showed negative associations with VAP and positive correlations with higher PaO_₂_/FiO_₂_ values, whereas *Escherichia-Shigella* and *Peptoniphilus* were associated with both VAP occurrence and lower PaO_₂_/FiO_₂_ ratios. Importantly, taxa associated with VAP onset were not always the same as those linked to impaired respiratory function, underscoring the complexity of host–microbiota interactions during critical illness.

This study should be interpreted within the context of the multifactorial pathogenesis of ventilator-associated pneumonia, in which microbial composition, host immune responses, and clinical interventions interact dynamically during mechanical ventilation. In line with previous observations, microbial alterations were detected prior to the clinical onset of VAP and were associated with both local immunological signals and progressive respiratory impairment.

The present work was designed to investigate early respiratory microbiota features associated with VAP occurrence and contemporaneous respiratory dysfunction, as captured by the PaO_₂_/FiO_₂_ ratio, rather than long-term or global ICU outcomes. Clinical endpoints such as mortality, ICU length of stay, mechanical ventilation duration, or organ failure are influenced by multiple interdependent factors in critically ill patients and require dedicated study designs and multivariable frameworks to be assessed without overinterpretation.

Several methodological considerations should therefore be acknowledged. Although patients were matched on center, indication for intubation, sex, and age, global illness severity was not explicitly incorporated as a covariate in microbiota-based multivariable analyses. Severity scores such as APACHE II and GCS did not differ significantly between VAP and no-VAP patients within the matched microbiota subset, suggesting a comparable baseline clinical burden; however, residual confounding by disease severity cannot be fully excluded. In addition, other clinically relevant factors, including comorbidities, ventilatory parameters, sedation strategies, aspiration risk, and hemodynamic instability, were not jointly modeled. These variables are highly interrelated in the ICU setting and may contribute to both microbial community variation and VAP susceptibility, underscoring the need for study designs tailored to high-dimensional clinical-microbiome integration. Additional limitations of this study include the use of 16S rRNA gene sequencing, which restricted taxonomic resolution to the genus level and therefore requires cautious interpretation of potentially pathogenic taxa, and the exploratory nature of cytokine-microbiota analyses, which were limited to a subset of patients. Baseline sampling was performed as close as possible to the time of intubation, within 24 hours, but the exact timing was not uniform across all patients, potentially contributing to additional variability in early microbiota profiles. Although antibiotic exposure prior to enrollment was accounted for as a categorical variable, the limited sample size precluded a more granular assessment of the impact of antibiotic class, duration, or cumulative exposure on microbial community structure. Furthermore, respiratory microbiota profiling relied on endotracheal aspirates rather than bronchoalveolar lavage, antibiotic exposure prior to enrollment was permitted within a defined time window, and the cohort was derived from a single country and predominantly included patients intubated for neurological indications.

At the same time, the study is strengthened by its multicenter longitudinal design, matched case–control framework, simultaneous sampling of upper and lower airways, and the availability of pre-infection time points, which remain rare in VAP microbiota research. Although immediate clinical translation is premature, the identification of bacterial patterns associated with both VAP susceptibility and respiratory dysfunction supports the rationale for future studies integrating microbiota data with clinical severity and outcome measures, with the ultimate goal of informing microbiota-aware risk stratification or monitoring strategies in the ICU, potentially supported by shotgun metagenomic and metatranscriptomic approaches to refine functional and causal interpretations.

## Data Availability

Raw Italian 16S rRNA gene sequencing data are available through the SRA under the study accession number PRJNA1215034. Clinical data used in this study are derived from the PULMIVAP observational cohort. These data are not publicly available due to patient confidentiality and institutional data-sharing policies. However, they may be made available upon formal request to the Principal Investigator of the PULMIVAP study (laura.alagna@policlinico.mi.it), including a clear description of the intended use and analysis plan.

## SUPPLEMENTAL MATERIAL

Figure S1 spectrum.03193-25-s0001.tif Study design, inclusion criteria, and sampling workflow of the PULMIVAP study.

Figure S2 spectrum.03193-25-s0002.tif Temporal stability and compartmental overlap of respiratory microbiota.

Figure S3 spectrum.03193-25-s0003.tif Cytokine profiles during mechanical ventilation in VAP and no-VAP patients.

Supplemental materials spectrum.03193-25-s0004.docx Supplemental methods and legends for Figures S1 to S3 and Tables S1 to S7.

Supplemental tables spectrum.03193-25-s0005.xlsx Tables S1 to S7.

ASM does not own the copyrights to Supplemental Material that may be linked to, or accessed through, an article. The authors have granted ASM a non-exclusive, world-wide license to publish the Supplemental Material files. Please contact the corresponding author directly for reuse.
