# Supplementary figures and images for: Microbial signatures and host immune responses associated with the development of ventilator-associated pneumonia among patients with neurological injuries
Source: Microbiol Spectr. 2026 Mar 23;14(5):e03193-25. doi: 10.1128/spectrum.03193-25 (PMC13141929; doi:10.1128/spectrum.03193-25)

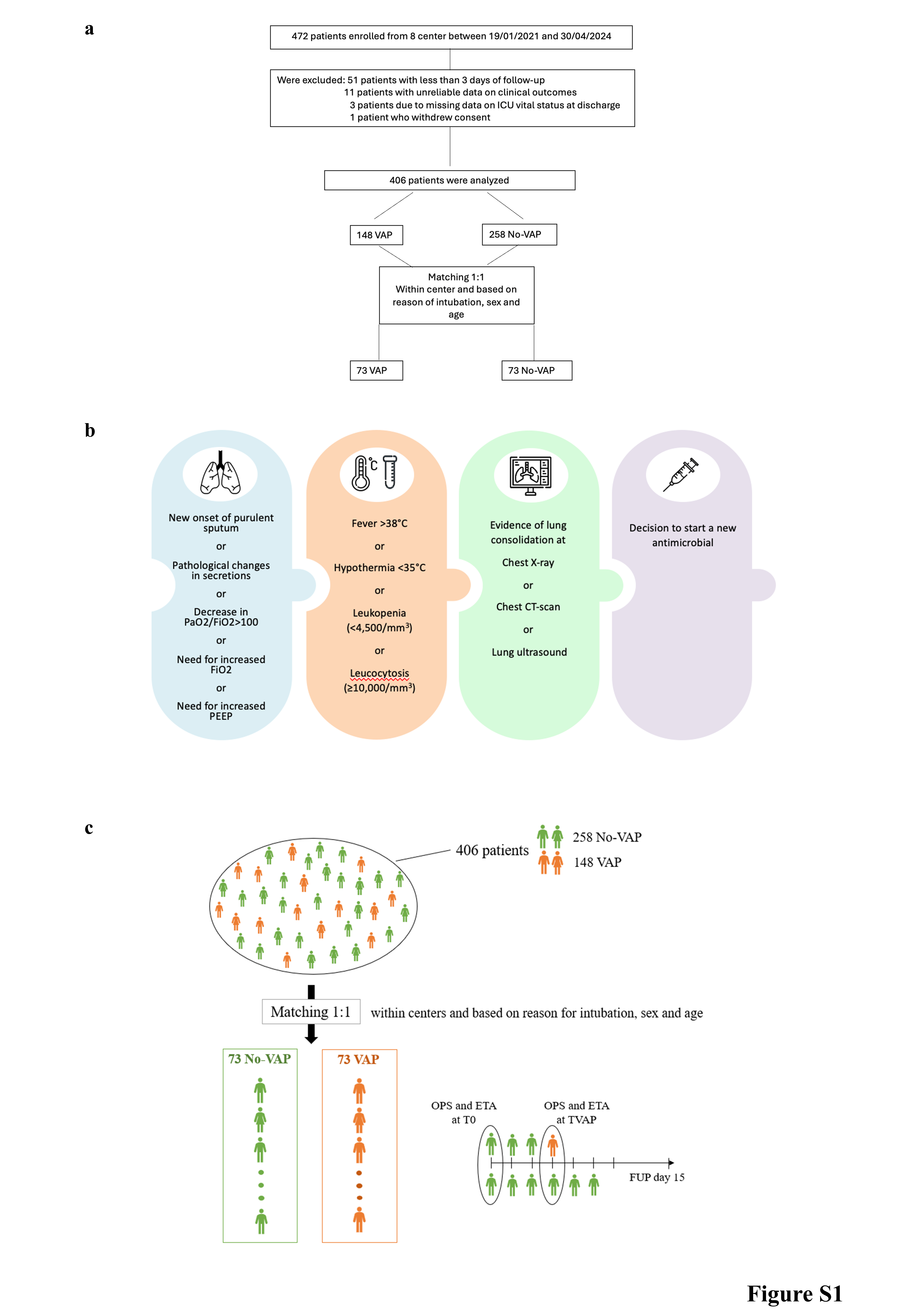

Supplement: Figure S1 — Study design, inclusion criteria, and sampling workflow of the PULMIVAP study. [file spectrum.03193-25-s0001.tif]

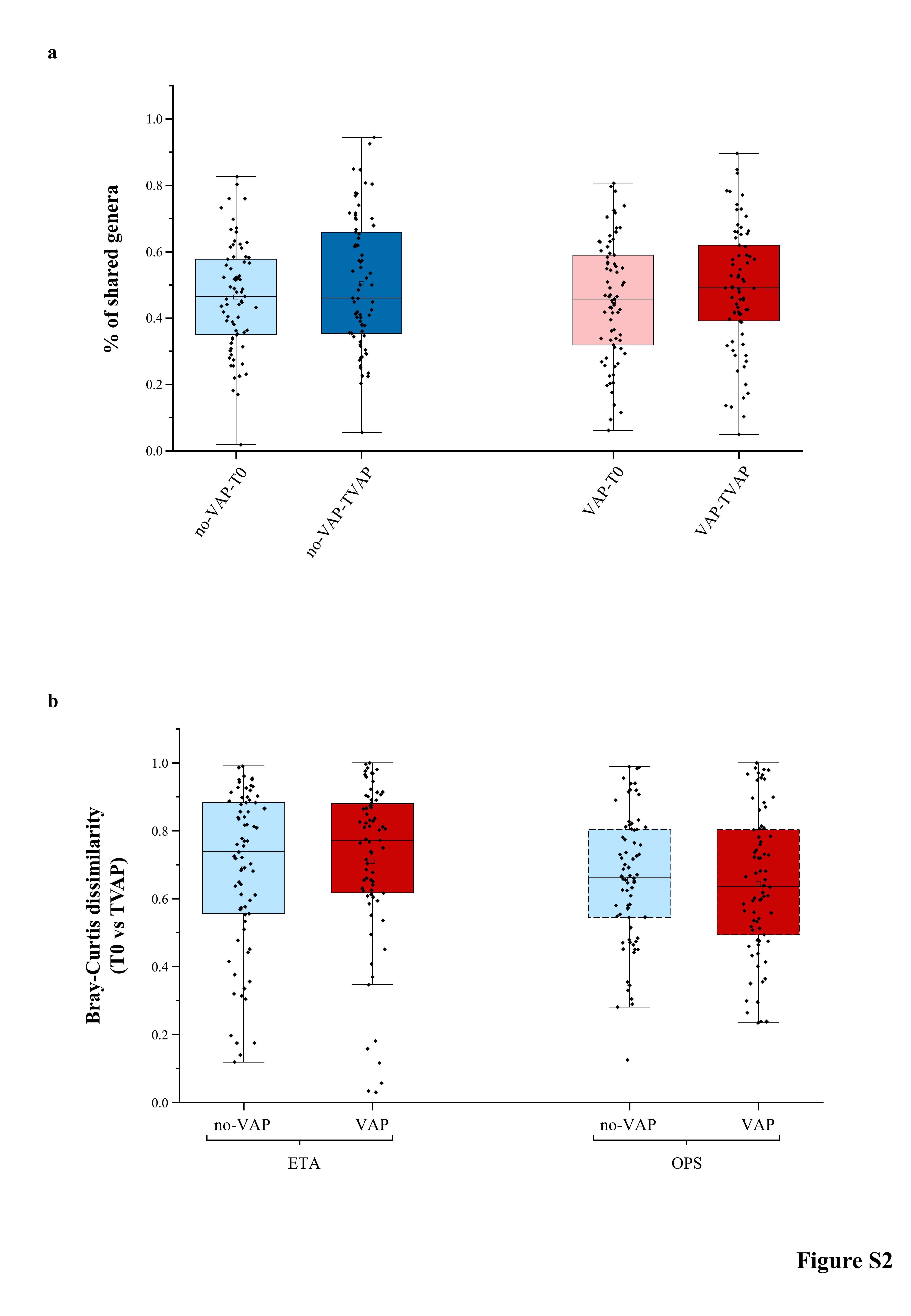

Supplement: Figure S2 — Temporal stability and compartmental overlap of respiratory microbiota. [file spectrum.03193-25-s0002.tif]

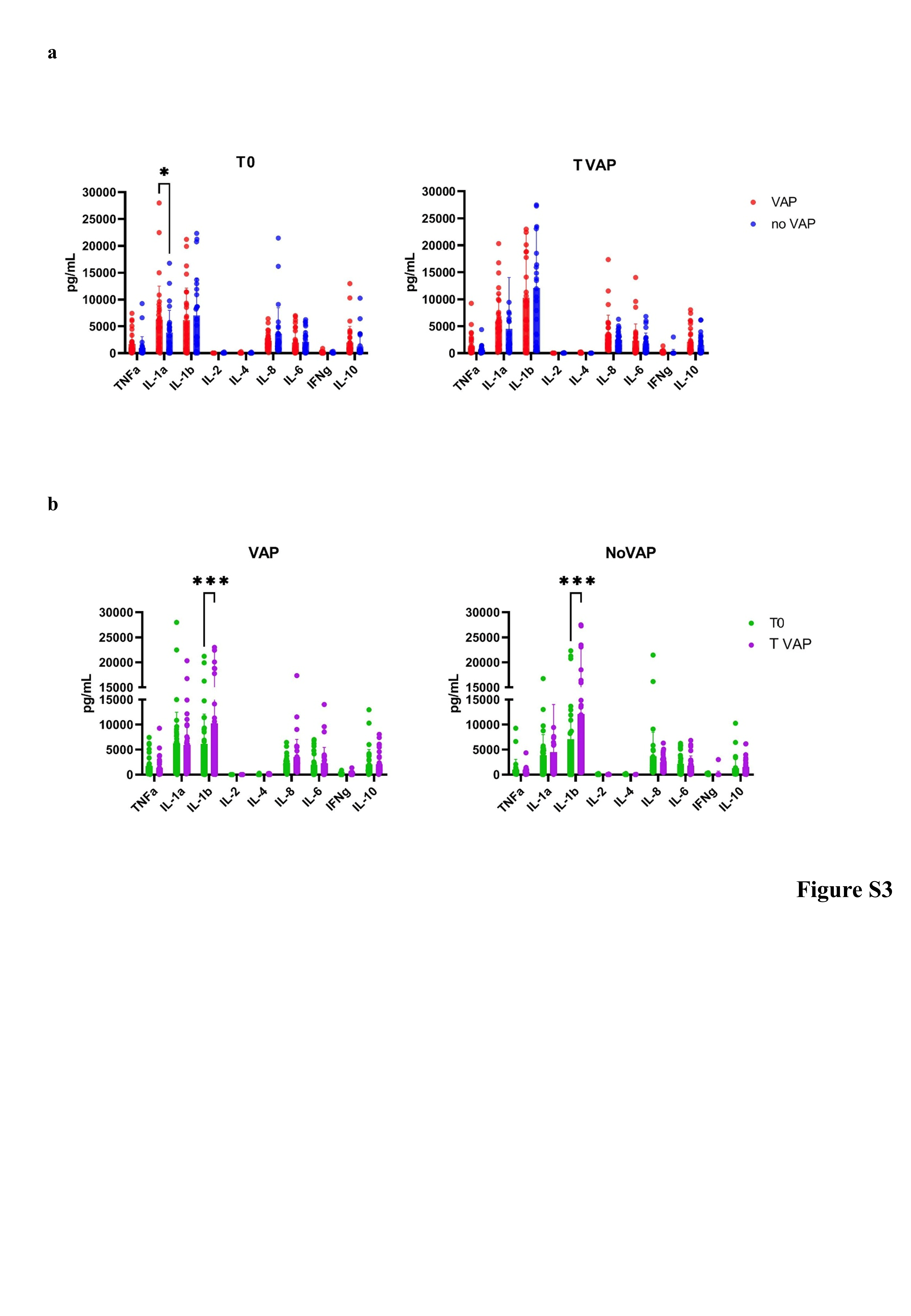

Supplement: Figure S3 — Cytokine profiles during mechanical ventilation in VAP and no-VAP patients. [file spectrum.03193-25-s0003.tif]
