# Supplementary material for: Microbial signatures and host immune responses associated with the development of ventilator-associated pneumonia among patients with neurological injuries
Source: Microbiol Spectr. 2026 Mar 23;14(5):e03193-25. doi: 10.1128/spectrum.03193-25 (PMC13141929; doi:10.1128/spectrum.03193-25)
Supplement: Supplemental materials — Supplemental methods and legends for Figures S1 to S3 and Tables S1 to S7. [file spectrum.03193-25-s0004.docx]

**Supplementary Materials**

**Materials and methods section**

**List of participating ICU centers**

Foundation IRCCS Ca' Granda Ospedale Maggiore Policlinico (Milan, Italy), Fondazione IRCCS San Gerardo dei Tintori (Monza, Italy), Ospedale Maggiore della Carità (Novara, Italy), ASST Spedali Civili di Brescia (Brescia, Italy), ASST Lecco A. Manzoni Hospital (Lecco, Italy), ASST GOM Niguarda (Milan, Italy), and University Hospital of Padova (Padova, Italy), Azienda Ospedaliero Universitaria di Parma (Parma, Italy).

**Reason for intubation**

Neurological reasons: airway protection for neurological causes (i.e., cerebrovascular accident, neurologic disease, brain trauma, brain surgery, etc.)

Non-neurological reason: airway protection for non-neurological causes (i.e., abdomen-thorax surgery, septic shock without lung involvement, multiple-trauma, anaphylaxis, etc.)

**Figures and tables legend (see Excel file and supplementary figure)**

**Table S1.** Cumulative incidence and 95% confidence intervals of first VAP, in a cohort of patients (pts) with less than 3 days of antibiotic therapy before MV and followed up to 15 days of intubation, overall and by Center. PULMIVAP Study (2021-2024).

**Table S2.** Setting and clinical characteristics of VAP and no-VAP patients, in the group of 73 VAP and 73 no-VAP patients.

**Table S3.** Outcome of matched patients (VAP and no-VAP)

**Table S4.** Setting and clinical characteristics of VAP and No-VAP patients in the PULMIVAP study (2021-2024), with VAP diagnosis according to clinical judgment

**Table S5.** DNA sequencing, data filtering, and metadata summary.

**Table S6.** Relative abundance of bacterial genera identified across all samples.

**Table S7.** Average relative abundance of bacterial genera per microbial cluster.

**Figure S1.** Study design, inclusion criteria, and sampling workflow of the PULMIVAP study. Panel a shows the VAP diagnostic criteria used in the PULMIVAP study. In detail, ventilator-associated pneumonia was considered in the presence of a worsening in ≥1 of the following domains: Inflammatory parameters (e.g., fever and/or white blood cell count), Radiologic infiltrates. Respiratory impairment (e.g., PaO₂/FiO₂ decline, increased FiO₂ requirement, or increased PEEP). The initiation of new antimicrobial therapy targeting pneumonia was recorded as a key indicator of a confirmed clinical diagnosis. Microbiological confirmation was obtained when endotracheal aspirate cultures showed bacterial growth ≥10⁴ CFU/mL within 3 days of diagnosis. For each component (temperature, white blood cell count, imaging findings, sputum characteristics, and oxygenation/ventilatory changes), the worst daily value was recorded. Panel b depicts the matching procedure and sampling strategy applied to define VAP and no-VAP patient groups. Panel c illustrates the study flow-chart.

**Figure S2.** Temporal stability and compartmental overlap of respiratory microbiota. Panels a and b show box-and-whisker plots of the percentage of shared microbial genera and Bray-Curtis dissimilarity between each sample at T0 and TVAP, respectively. Boxes represent interquartile ranges, whiskers indicate 1.5× IQR, and black lines denote medians.

**Figure S3.** Cytokine profiles during mechanical ventilation in VAP and no-VAP patients. Panels a and b show cytokines analysis among VAP and no-VAP patients during intubation. In detail, panel a indicates a comparison of cytokines expression at intubation (T0) and T-VAP between VAP and no-VAP. Panel b shows cytokines expression during MV (T0 and T-VAP) among VAP and no-VAP patients.
